# FAMILY CAREGIVER READINESS: RACIAL DIFFERENCES AND RELIABILITY OF THE CAREGIVER READINESS SCALE

**DOI:** 10.1093/geroni/igz038.649

**Published:** 2019-11-08

**Authors:** Katherine A Marx, Katherine A Marx, Lauren J Parker, Jin Huang, Daniel Scerpella, Karen Rose, Catherine V Piersol, Laura N Gitlin

**Affiliations:** 1 Johns Hopkins University, Baltimore, Maryland, United States; 2 Widener University, Chester, Pennsylvania, United States; 3 Thomas Jefferson University, Philadelphia, Pennsylvania, United States; 4 Drexel University, Philadelphia, Pennsylvania, United States

## Abstract

The success of any intervention for caregivers of persons with dementia is dependent on the caregiver’s readiness to enact the strategies. This presentation explores the reliability of the new 17-item Caregiver Readiness Scale (CRS) and also to examine the differences by race in readiness. Participants were caregivers in the Dementia Behavior Study who completed the CRS at baseline (n=129). Caregivers were on average 65.8 years old (sd = 12.2, range 28-88), the majority reported their race as non-Hispanic white (64.3%, n=83) and 33.4% (n=43) reported their race as African American or other. The average CRS score was 57.63 (sd=5.72, 40-68) (α=0.73). The only significant interaction with race was negative communication (p=0.026) with negative communication scores having little effect on readiness in whites, but in non-white caregivers, there was an inverse relationship. Knowing the caregiver’s level of readiness and communication style may help improve the acceptability and success of an intervention.

